# Global Health: A Successful Context for Precollege Training and Advocacy

**DOI:** 10.1371/journal.pone.0013814

**Published:** 2010-11-03

**Authors:** Ana L. Gervassi, Laura J. Collins, Theresa B. Britschgi

**Affiliations:** 1 BioQuest, Seattle Biomedical Research Institute, Seattle, Washington, United States of America; 2 Center for Research and Learning, Snohomish, Washington, United States of America; Kenya Medical Research Institute, Kenya

## Abstract

Despite a flourishing biomedical and global health industry [1] too few of Washington state's precollege students are aware of this growing sector and emerging ideas on bacteria, fungi, parasites and viruses. Against the backdrop of numerous reports regarding declining precollege student interest in science [2], a precollege program was envisioned at Seattle Biomedical Research Institute (as of 2010, Seattle BioMed) to increase youth engagement in biomedical research and global health, increase community interest in infectious diseases and mobilize a future biomedical workforce. Since 2005, 169 rising high school juniors have participated in the BioQuest Academy precollege immersion program at Seattle BioMed. Assembling in groups of 12, students conduct laboratory experiments (e.g., anopheline mosquito dissection, gene expression informed tuberculosis drug design and optimizing HIV immunization strategies) related to global health alongside practicing scientific mentors, all within the footprint the institute. Laudable short-term impacts of the program include positive influences on student interest in global health (as seen in the students' subsequent school projects and their participation in Seattle BioMed community events), biomedical careers and graduate school (e.g., 16.9% of teens departing 2008–2009 Academy report revised goals of attaining a doctorate rather than a baccalaureate diploma). Long-term, 97% of alumni (2005–2008) are attending postsecondary schools throughout North America; eight graduates have already published scientific articles in peer-reviewed journals and/or presented their scientific data at national and international meetings, and 26 have been retained by Seattle BioMed researchers as compensated technicians and interns. Providing precollege students with structured access to practicing scientists and authentic research environments within the context of advancing global health has been a robust means of both building a future pool of talented leaders and engaged citizenry and increasing the visibility of health disparities within the community.

## Introduction

Teens from racial and ethnic backgrounds underrepresented in the biomedical sciences consider a myriad of intrinsic and extrinsic factors (peers, family, educational foundation and long term compensation) as they approach college age [3]. With limited role models from scientific fields reflecting their individual race, ethnic, and/or economic status appearing in their classrooms, neighborhoods or social media pages, these students' abilities to envision a world advanced by their own intellectual capital and innovation are significantly hampered [4].

Global Health has been defined as “the area of study, research and practice that places a priority on improving health and achieving equity in health for all people worldwide” [5]. The recent rise in media coverage of the wide-ranging and devastating impacts of global health, and in particular global infectious diseases, has illuminated global scourges as well as the opportunities for progress [6]. Seattle Biomedical Research Institute (as of 2010 as Seattle BioMed) is the largest independent, non-profit organization in the United States that since 1975 has focused solely on disease discovery research in five infectious disease areas, including HIV/AIDS, Malaria, microbial pathogens, trypanosomatids and tuberculosis. Successes with University of Washington Pathobiology graduate student training and the numerous national reports on declining American student science and math achievement [7] motivated Seattle BioMed leaders to launch the BioQuest precollege outreach program within the footprint of its downtown research facility [8]. Informed by the Harvard Family Research Project's best practices for out-of-school programs [9], BioQuest includes outreach programs that train teachers, students, scientists and the general public. The mission of BioQuest is to enhance community global health awareness and address projected gaps in our research laboratories. The BioQuest Academy immersion program is our most intensive student offering, with high mentorship, instructional tactics and curriculum that link the activities of biomedical researchers and public health specialists to the course work students need to matriculate as well as enter college and biomedical careers. Five years of feedback and evaluation data obtained from the 169 enrolled students indicate that intentionally designed precollege training programs that feature real-world contexts and strong mentorship influence student long-term college, career and advocacy activities and increase local interest in biomedical research.

## Methods

### Ethics Statement

This study was conducted according to the principles expressed in the Declaration of Helsinki. Seattle BioMed researchers received written informed consent from 2005-07 BioQuest Academy parents to have their minor children participate in the 2005-07 Academy sessions and for the collection and subsequent analysis of students' verbal and written responses to pre-, post- and focus group tools. With federal grant support, the revised 2008-09 study protocol was approved by the Western Institutional Review Board (WIRB #20071499) to also include permission to collect and analyze student interview responses. Taken together, all study participants and/or their legal guardians have provided written or tape recorded (by the members of the Center for Research and Learning) informed consent for the collection and subsequent analysis of student verbal and written responses.

### Recruitment and Enrollment

High school students from increasingly larger pools of racially diverse applicants from public, private and parochial schools across Washington State learn of the Academy and the online application process through multiple avenues. Most students learn of the program by attending half-day field trips to Seattle BioMed with their teacher, while other applicants get program information by targeted recruitment. Since 2005, a total of 169 high school juniors, in groups of 10–12, have enrolled in the BioQuest Academy from increasingly larger pools of applicants (see Table 1 for a review of the applicant and cohort pools). The composition of the Academy cohort is evolving. A 2008 Science Education Partnership Award (SEPA) from the National Center for Research and Resources at the National Institutes of Health greatly enhanced our ability to recruit more students from underserved racial minority groups (URM, see Table 1). Strategies that encourage the greater participation of URM students include offering student stipends, BioQuest staff visits to target Washington State high schools, solicitations via Hispanic TV and radio stations and via alliances with local organizations that support low income and/or minority students. Online application question prompts have been developed over the past five years to query student foundational interest in research (“describe your favorite lab experiment and how you might alter or improve it if you could repeat it”) and global health (“what college and career plans do you have that will allow you to improve global health”). Eligible applicants provided a supporting teacher nomination wherein teachers confirm the student age and their last science grade. A panel of staff, former students and community partners review each application. Those students that are best able to relay strong empathy and curiosity to reviewers are selected for enrollment. Parents of accepted minors, along with all adult participants, complete an IRB-approved release form (Western Institutional Review Board), acknowledging their awareness of the Academy as a human behavior investigation wherein we are assessing the short- and long- term impact of the program on participants.

**Table 1:** 2005-09 cohort description and post-secondary trajectories.

Academy Session	2005	2006	2007	2008	2009
**Total # of Applicants**	61	60	60	115	237
**Total # of students enrolled**	29	29	36	26	48
**% Minority representation in Academy sessions**	14%	14%	8%	42%	55%
**% Academy Seniors students entering 4-Year Colleges**	93%	100%	97%	92%	96%
**Undergraduate persistence (as of June 2010)**	90%	97%	97%	92%	N/A
**Cohort status (Summer 2010)**	College graduates or 5th year seniors	College seniors	College juniors	College sophomores	College freshman

Students enrolled in 2005-7 sessions participated in a 30 hour program. Students, recruited through year-long enrollment activities, participated in a 60 hour program.

### Program Description

**Figure 1:**
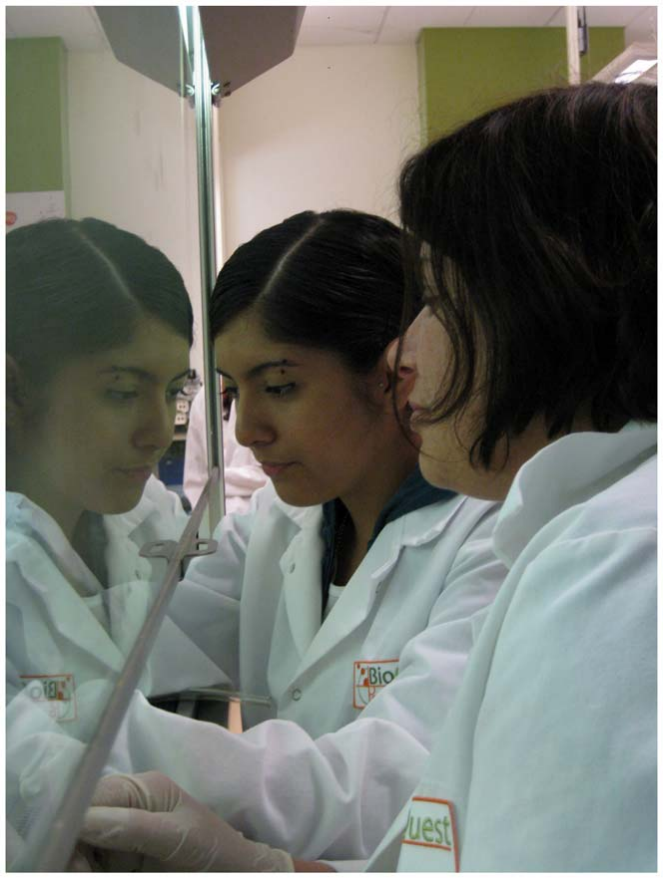
BioQuest Academy student extracts *M. smegmatis* mRNA with mentor assistance. Credit: Grace Itaya.

The BioQuest Academy largely takes place within a biosafety-level-2-rated dedicated education laboratory located within Seattle BioMed. Institute and partner organization scientists mentor the students under the direction of BioQuest staff—who are also veterans of the biotechnical and biomedical industry (see student and mentor in action in Figure 1. Photograph provided with permission of subjects). Over the course of the 30 or 60 hour program students follow a rigorous curriculum that features national science standards embedded in methodologies practiced in infectious disease research (SEPA support allowed staff to double the length of the program in 2008). Seattle BioMed investigators have donated unique resources such as serum from animals immunized with HIV gp120 protein and plasmid constructs containing cloned *var2csa* gene segments procured from geographically disparate *Plasmodium* isolates [10]. All laboratory investigations, from *Anopheles* mosquito dissection to gene expression analysis of Mycobacterial latency, are listed in Table 2. Only 2008-09 students performed the Team activities in the table. Each investigation features guest expert lectures and a variety of related visual media resources (e.g., movies and websites). Students spend their final Academy session days preparing project presentations, Facebook™ networking with peers and staff, drafting college entrance essays and updating their individual resumes with newly learned skills. After sessions end, BioQuest staff support departing student success during their senior year in high school. While each student varies in their needs, the staff provides anywhere from 5–30 hours of mentoring by hosting SAT test study sessions, advising senior projects and university applications (e.g., writing letters of recommendation and offering editorial assistance for application essays and resumes) and providing professional references for laboratory research positions.

**Table 2:** BioQuest Academy 2008-09 laboratory activities.

Format	Name	Activity
Paired	Natural Defenses	Examinations of normal flora through microbiological culture, gram stain analysis and yeast-targeting PCR reactions.
Paired	Disease Transmission	Pathogen transmission demonstrations using Glo-Germ sanitary gel.
Paired	Introduction to Immunology	Ficoll-hypaque separation and ELISA assays punctuate discussions of the immune system, HIV and epidemiology.
Paired	Malaria	*Anopheles* mosquito dissections.
Paired	Mosquitoes and Microarrays	Cartoon microarray patterns are linked to different mosquito pathologies.
Paired	Drug Assay Optimization	Candida E-test® plates and bacterial hydrolase assays are linked to drug resistance and drug development.
Paired	Tuberculosis Case Study	A fictitious tuberculosis case study is linked to DNA gel electrophoresis patterns.
Team	HIV Vaccine Characterization	Investigating rabbit immune response to diverse HIV immunization strategies (antibody affinity purification, ELISA and Western analysis).
Team	Malaria *Var* Gene Analysis	Bioinformatic analysis of geographically diverse cloned *Var* gene subunits; relevancy to malaria vaccine development.
Team	Drug Discovery through *M. smegmatis* Gene Expression Analysis	Students prepare and analyze microarray data prepared from *M. smegmatis* mRNA; relevancy to tuberculosis drug discovery.
Solo	Student Disease Research	Students present their pathogen internet research.
Solo	College Preparatory Curriculum	Students identify the ideal universities (including application criteria) where they may continue to learn more infectious disease research and global health.

Alignment of BioQuest Academy resources to Washington State high school science standards has been confirmed by external curriculum consultants [17].

### Evaluation

A well-defined evaluation and research plan has given Seattle BioMed staff the information they need to make data-based decisions to improve delivery, foster accountability and determine whether the program is achieving its training and awareness goals. Short term student impacts were discerned from data collected at the time of the Academy. Students completed survey tools at the start and at the end of the Academy. Test items included content knowledge, as well as attitudes toward science, awareness of biomedical careers and response to specific Academy activities. Data from the tests were entered into SPSS (Statistical Package for the Social Sciences) and analyzed for means, standard deviations and statistically significant differences. As a complement to the student feedback collected proximal to the Academy, students also participated in follow-up interviews during their first year of college concerning Academy outcomes and activities, as well as future coursework, career options and connections with Seattle BioMed. The open nature of the interview allowed the students to expand upon their experiences during the Academy and reflect on the impact that the program had on their college coursework and career aspirations. Longitudinal tracking was additionally supported by direct interaction by email, phone and Facebook™-mediated discourse between program staff and students. Data collected during participant interviews were qualitatively analyzed through constant comparison methods to identify themes and major categories of response. BioQuest staff collected and analyzed the number of public inquiries made with regards to BioQuest Academy programming, as well as teacher and family attendance at Seattle BioMed scientific lectures and BioQuest Academy website visitation metrics as a reflection of community interest infectious disease.

## Results

**Figure 2:**
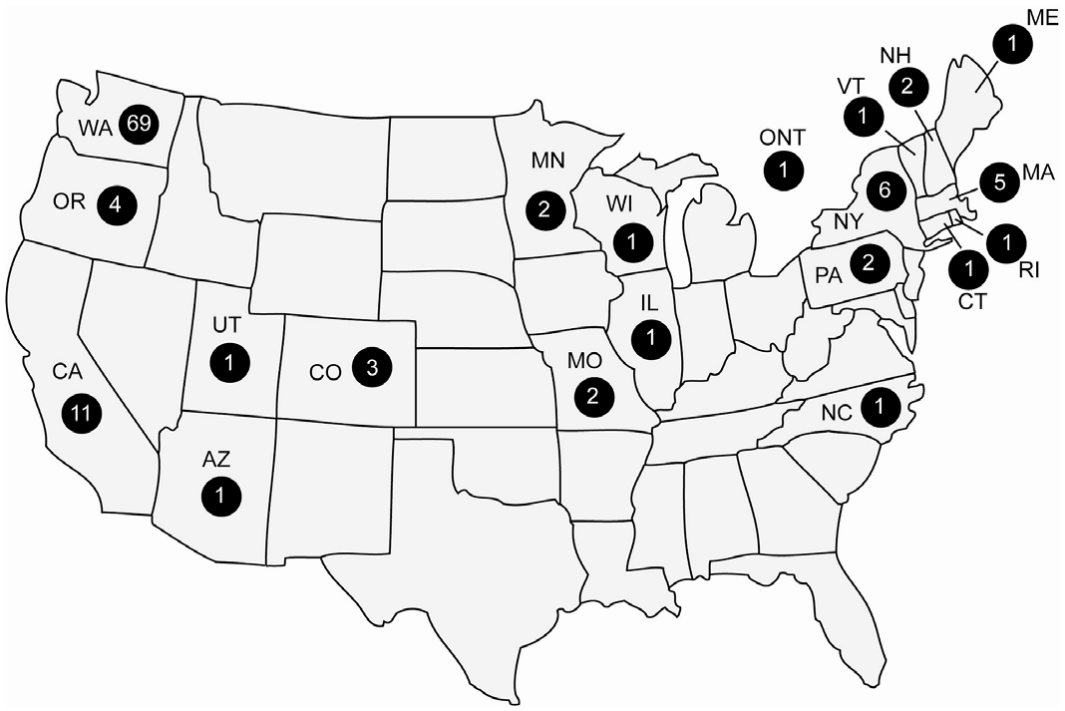
College location and quantity of Academy graduates in each state. Credit: Marissa Vignali.

Seattle BioMed leadership launched a mosaic of precollege outreach modules as the BioQuest Program in 2004 to address to anticipated workforce challenges and to increase community engagement around neglected diseases of global concern. Since launching the program in 2005 BioQuest modules (e.g., half-day job shadows, teacher professional development programs, summer internships and the BioQuest Academy) involve the participation of 100–120 Seattle BioMed scientists per year and deliver activities and curriculum of varying duration and rigor all relating to Seattle BioMed research focus. Each year, greater numbers of students apply for our programs (see Table 1), 10–15% of guests attending Seattle BioMed public scientific lectures are BioQuest teachers and families and web traffic to BioQuest has grown exponentially (e.g., last year 10,100 accessed BioQuest content and multi-media). Academy elements—including hands-on lab activities in an authentic research organization, resume-building experience conducting college level lab techniques, and a pre-existing interest in infectious diseases—have attracted 169 precollege students of increasing diversity to attend the Academy since 2005. Four years of quantitative and qualitative evaluation collected by the Center for Research and Learning demonstrates that over the duration of the Academy enrolled students report statistically significant gains in students' awareness of global infectious diseases as well as their understanding of the expertise and training needed in biomedical fields (see Table 3). For example, mentor conversations helped students realize that attaining global health careers might require additional education, to the extent that students planning to obtain their doctorate diploma increased in 2008 from 34.6% (entry) to 52% (departure) and in 2009 from 30.6% (entry) to 46.9% (departure). All students (100%) who have attended Academies indicate in exit surveys that they would recommend the program as a valuable experience to other students. Through regular contact, by email, phone calls and even social media networks, BioQuest staff has been able to track the long term progression of 168 of the 169 Academy graduates. As of April 2010, 119 of the Academy graduates have graduated from high school. Over 90% of the five Academy cohorts have subsequently enrolled as a freshman in a four-year university (see Table 1), attending schools across North America (see Figure 2 for university locations). Academy students, from both targeted (2005-7) and untargeted cohorts (2008-9), enroll and persist in undergraduate education programs at higher than national trends of 27.8% [11]. This year, 93% of the first year cohort (2005) will complete undergraduate studies with supporting majors in public health, pre-medicine, nursing, bioengineering, biochemistry, neurobiology, information science; as well as linguistics, economics and political science. Eight students have conducted university research investigations, resulting in peer-review level publications and poster presentations [12], [13] and 15.4% of students (26 graduates) have joined research programs at Seattle BioMed as compensated research interns. In addition to performing undergraduate research, nearly 20 graduates have demonstrated their passion for advancing international health by participating in university international studies programs in countries such as Australia, Belize, Benin, Bermuda, Ecuador, Haiti and Uganda. Phone interviews with Academy graduates (16 months after departing their sessions) indicate that all queried students felt strongly that the engaging experiences of the program influenced how they view the importance of science, sparked their interest in infectious disease and helped them better visualize their training and career futures. Graduates stated in interviews that Academy experiences inspired their high school senior project topics, applications and acceptance to colleges of their choice, informed their selection of colleges, majors and lab internships and future job prospects (including employment at Seattle BioMed).

**Table 3:** 2005-09 Academy student pre- and post-perceptions.

Please tell us how much you know about the following categories:							
Items		Hardly Anything (1)	Not Too Much (2)	Average (3)	Above Average (4)	Mean	Standard Deviation
**Global Health**	Pre	4.2%	35.5%	51.2%	9.0%	2.65	0.70
	Post*	0.0%	0.6%	11.3%	88.1%	3.88	0.35
**Infectious Diseases**	Pre	4.2%	31.3%	51.8%	12.7%	2.73	0.73
	Post*	0.0%	0.6%	8.9%	90.5%	3.90	0.32
**Laboratory Safety**	Pre	1.2%	9.0%	55.4%	34.3%	3.23	0.66
	Post*	0.0%	0.0%	8.9%	91.1%	3.91	0.29
**Basic Lab Techniques**	Pre	4.8%	21.1%	46.4%	27.7%	2.97	0.83
	Post*	0.0%	0.0%	11.9%	88.1%	3.88	0.32
**Completing Lab Notebooks**	Pre	2.4%	17.6%	47.9%	32.1%	3.10	0.77
	Post*	0.0%	1.8%	40.5%	57.7%	3.56	0.53
**Careers in Biomedical Research**	Pre	16.3%	48.2%	31.3%	4.2%	2.23	0.77

Using quasi-experimental methods of pre-post testing each year, students have responded to a variety of questions that reveal students' significant gains in global health and biomedical research content between pre- and post- program responses, * =  p<.001.

## Discussion

Seattle BioMed training and advocacy objectives, vital to the mission of Seattle BioMed as well as national biomedical workforce diversification goals, have been advanced by the development of the Academy precollege program. The results of data gathering instruments and ongoing dialog with graduates demonstrate that the BioQuest Academy immersion program impacts students' attitudes toward science and significantly increases students' knowledge of infectious disease and biomedical careers. Targeted recruitment strategies are attracting increasing numbers of students from a spectrum of ethnicities and races to apply and participate. Student longitudinal trends related to college enrollment, college persistence and overall interest in biomedical and health-related fields are consistently higher than national trends, regardless of an increasingly diverse student cohort, suggesting that Academy students depart better prepared to successfully navigate post-secondary endeavors (e.g., college entrance SAT tests, college entrance and undergraduate persistence) and subsequently address the long term labor needs of the biomedical community. As graduates apply for and participate in Seattle BioMed discovery research programs as undergraduates, they frequently state that their experience in the Academy as the place where their affinity for the Institute mission began and the reason why they sought employment and volunteer opportunities at Seattle BioMed. In addition to advancing Institute training goals, colleagues from our clinical trials teams appreciate the exposure that their programs get through BioQuest. Last year alone, over 600 students and teachers who accessed BioQuest programming were shown authentic recruitment and regulatory content related to the Institute's genetically attenuated parasite vaccine approach. Interactive and educational conversations through BioQuest resulted in numerous inquiries to the Seattle BioMed Malaria Clinical Trials Center website and staff regarding opportunities for trials enrollment [unpublished statistics]. Data and ongoing dialog with graduates demonstrate that providing precollege students with structured access to practicing scientists and authentic research environments within the context of advancing global health has proven to be a robust means of both increasing the visibility of health disparities. As graduates integrate infectious disease content into their community service projects or declare their predilection for global health related post-secondary learning and training opportunities they are influencing the development of baccalaureate programming. Influential community stakeholders from across the public and private sectors [14], [15]; and as far as Delhi, Ghana, Kenya, Peru and Queensland have requested copies of our evaluation tools and curriculum [16].

Since launching its doors in 1976 Seattle BioMed has included post-secondary, scientific training opportunities for students. Since 2005, and the launch of the BioQuest Academy, increasing numbers of Seattle BioMed investigators have hired Academy students to join their research teams as undergraduates (e.g., over ten Academy graduates will contribute to the research mission of Seattle BioMed in 2010). Providing precollege students with structured access to practicing scientists and authentic research environments within the context of advancing global health has proven to be a robust means of both increasing the visibility of health disparities, refining adolescent preconceptions of research and producing scientists and leaders who can passionately address the challenges of global disease and inequity and, one day, deliver a healthier world.
